# AltitudeOmics: Baroreflex Sensitivity During Acclimatization to 5,260 m

**DOI:** 10.3389/fphys.2018.00767

**Published:** 2018-06-21

**Authors:** Nicolas Bourdillon, Sasan Yazdani, Andrew W. Subudhi, Andrew T. Lovering, Robert C. Roach, Jean-Marc Vesin, Bengt Kayser

**Affiliations:** ^1^Institute of Sports Sciences of the University of Lausanne, Lausanne, Switzerland; ^2^Applied Signal Processing Group, Ecole Polytechnique Fédérale de Lausanne, Lausanne, Switzerland; ^3^Department of Biology, University of Colorado, Colorado Springs, CO, United States; ^4^Altitude Research Center, Department of Medicine, University of Colorado Anschutz Medical Campus, Aurora, CO, United States; ^5^Department of Human Physiology, University of Oregon, Eugene, OR, United States

**Keywords:** altitude, BRS, baroreflex, hypoxia, acclimatization, CO_2_

## Abstract

**Introduction:** Baroreflex sensitivity (BRS) is essential to ensure rapid adjustment to variations in blood pressure (BP). Little is known concerning the adaptive responses of BRS during acclimatization to high altitude at rest and during exercise.

**Methods:** Twenty-one healthy sea-level residents were tested near sea level (SL, 130 m), the 1st (ALT1) and 16th day (ALT16) at 5,260 m using radial artery catheterization. BRS was calculated using the sequence method (direct interpretation of causal link between BP and heartrate). At rest, subjects breathed a hyperoxic mixture (250 mmHg O_2_, end tidal) to isolate the preponderance of CO_2_ chemoreceptors. End-tidal CO_2_ varied from 20 to 50 mmHg to assess peripheral chemoreflex. Rebreathing provoked incremental increase in CO_2_, increasing BP to assess baroreflex. During incremental cycling exercise to exhaustion, subjects breathed room air.

**Results:** Resting BRS decreased in ALT1 which was exacerbated in ALT16. This decrease in ALT1 was reversible upon additional inspired CO_2_, but not in ALT16. BRS decrease during exercise was greater and occurred at lower workloads in ALT1 compared to SL. At ALT16, this decrease returned toward SL values.

**Discussion/Conclusion:** This study is the first to report attenuated BRS in acute hypoxia, exacerbated in chronic hypoxia. In ALT1, hypocapnia triggered BRS reduction whilst in ALT16 resetting of chemoreceptor triggered BRS reduction. The exercise BRS resetting was impaired in ALT1 but normalized in ALT16. These BRS decreases indicate decreased control of BP and may explain deteriorations of cardiovascular status during exposure to high altitude.

## Introduction

High altitude hypoxia challenges blood pressure (BP) homeostasis in humans. In the short term, baroreceptor afferents principally counteract the stress of hypoxia on BP homeostasis by affecting the activity of the parasympathetic and sympathetic divisions of the autonomic nervous system (ANS). Hypoxemia induces vasodilation and therefore hypotension, which stimulates the baroreflex function. The baroreceptor-mediated sympathetic excitation in hypoxia results in an increase in set point (Halliwill and Minson, 2002) and a decrease in gain (Bernardi et al., 1998; Cooper et al., 2005), therefore affecting the BP regulation.

Baroreflex sensitivity (BRS) is a measure of baroreflex function where the faster the response to small changes in BP, the more sensitive the autonomic control of BP and the higher the BRS. The baroreceptors, located in the aortic arch and the carotid bodies, signal to the medulla via the cranial nerves IX and X (Cowley et al., 1973). The ANS also receives afferents from chemoreceptors, of which the peripheral chemoreceptors are sensitive to changes in arterial blood O_2_ and CO_2_ (pHa), whereas the central chemoreceptors, located in the CNS, are primarily sensitive to variations in CO_2_ (pHCSF), but not in O_2_, unless arterial O_2_ saturation (SaO_2_) falls below 50% (Dempsey et al., 2014; Smith et al., 2015). These baro- and chemo-reflex arcs coincide, so that sensory information regarding BP and arterial blood gas homeostasis converge in an integrative fashion (Vasquez et al., 1997). In humans, there is a negative relationship between the baro- and chemoreflexes; that is, baroreflex activation inhibits the chemoreflex and vice versa (Somers et al., 1991; Cooper et al., 2005). How these responses are ultimately integrated and expressed to regulate BP homeostasis at rest and exercise in acute and chronic hypoxia is largely unknown and is the focus of this report.

During exercise, increases in cardiac output (CO) and changes in systemic vascular resistance (SVR), renal and gastrointestinal vasoconstriction, along with working muscle vasodilatation, cause an increase in blood pressure that activates the arterial baroreceptors (Michelini et al., 2015). Yet, mean blood pressure during dynamic exercise only increases moderately, because there is a resetting of BRS to increased arterial pressures as a function of the intensity of the dynamic exercise (Bevegård and Shepherd, 1966; Eckberg et al., 1975; Pawelczyk and Raven, 1989; Joyner, 2006). BRS thus seems to be reset from rest to 75% of maximum oxygen consumption (Potts et al., 1993; Papelier et al., 1994). To the best of our knowledge, no study has reported BRS during dynamic exercise during an acclimatization process to high altitude in humans.

To reveal the role of changes in BRS over time in hypoxia on BP homeostasis, BRS was quantified with intra-arterial pressure measurements in humans during acute and chronic hypobaric hypoxia at rest and during exercise, and whilst breathing various O_2_ and CO_2_ mixtures. We hypothesized that (1) BRS would be attenuated in acute hypoxia and further decreased after acclimatization at rest and during exercise; (2) and that additional inspired CO_2_ would have distinct effects at sea level, and during acute through chronic hypoxia, that would indicate a resetting of BRS toward low PaCO2 values after acclimatization.

## Materials and methods

### Subject recruitment and screening

This study is part of the AltitudeOmics project (Subudhi et al., 2014). Twenty-one young, healthy, sea-level residents, average age 21, range 19–23 years, were recruited in the region of Eugene, Oregon, USA (130 m). Physical examinations and the U.S. Army Physical Fitness Test [APFT, push-ups, sit-ups, and a 3.2-km run (Knapik, 1989)] were performed to characterize health and fitness status. Exclusion criteria included being born at >1,500 m, having traveled to altitudes >1,000 m in the past 3 months (including air travel), using prescription medications, smoking, being pregnant or lactating, having a history of serious head injury (loss of consciousness), self or familial history of migraine, known hematologic or cardiovascular abnormality (e.g., sickle cell trait, cardiac arrhythmia), pulmonary function or diffusion capacity for carbon monoxide <90% of predicted, or failure to meet the minimal age/gender standards for the APFT (Knapik, 1989). Subjects' characteristics are summarized in Table 1, which is a reproduction from previously published AltitudeOmics results (Subudhi et al., 2014). Preliminary BRS results have been published as conference proceedings (Yazdani et al., 2016a,b). There is no further redundancy between the present data analysis and other publications from the AltitudeOmics project.

**Table 1:** General Subject Characteristics.

	**Sex**	**Age (years)**	**Height (cm)**	**Weight (kg)**	**BMI (kg/m^2^)**
Mean	12M/9F	20.8	175.8	69.7	22.4
SD	–	1.4	7.9	9.0	1.8

### Ethical approval

The study was approved by the institutional review boards of the University of Colorado and the University of Oregon and by the Human Research Protection Office of the US Department of Defense and was performed according to the Declaration of Helsinki. The subjects were informed about the procedures and risks and gave written consent prior to participation.

### Experimental design

Familiarization with the experimental procedures included a graded exercise test up to exhaustion ($\overset{{}^{\circ}}{\text{V}}$O_2p_ test) to assess the aerobic fitness of the subjects and to ensure that the inclusion criteria were met. After familiarization, the subjects underwent experimental trials near sea level (SL, 130 m; barometric pressure 749 mmHg) and on the 1st (ALT1) and 16th day (ALT16) at 5,260 m (barometric pressure 406 mmHg). For each subject, all ALT measurements were carried out around the same time of day to minimize any confounding effects of the circadian rhythm. During ascent (from 1,525 to 5,260 m) the subjects breathed supplemental oxygen (2 L/min, nasal cannula or mask). Administration of O_2_ was ceased just before ALT1 measurements. This ensured standardized acute exposure at ALT1 and minimized any influence of early acute mountain sickness (AMS) during ALT1. Likewise, no symptoms of AMS were observed at ALT16 because of successful acclimatization. An overview of the entire experimental design of the AltitudeOmics project is given elsewhere (Subudhi et al., 2014).

### Experimental protocol

Before entering the experimental room, the subjects laid down in a room dedicated to the insertion of an arterial catheter (20–22 gauge) into a radial artery (Arrow International, Reading, PA, USA) under local anesthesia (2% lidocaine). Arterial blood pressure was measured using this catheter and a calibrated pressure transducer (Deltran®, Utah Medical, UT, USA) connected to an amplifier (BP amp, ADInstruments, CO, USA). After ~30 min of instrumentation, the subjects underwent the resting protocol, followed by the exercise protocol.

### Resting protocol

**Figure 1:**
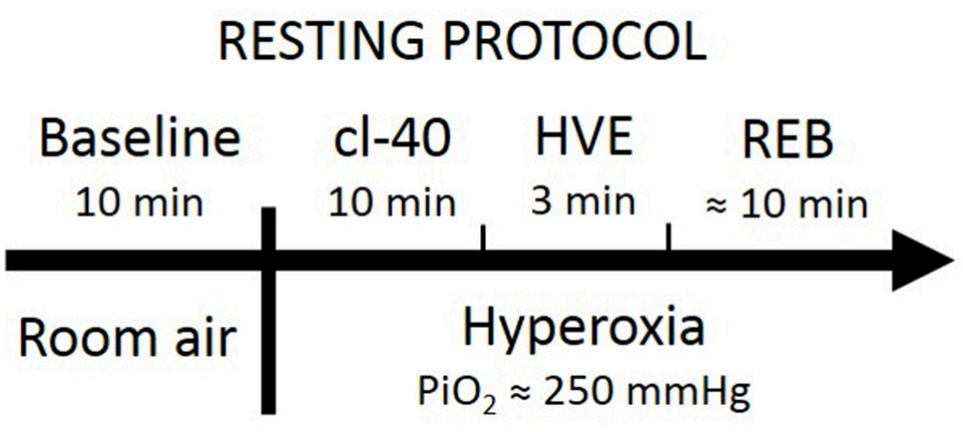
Block-diagram of the timeline for the resting protocol.

Following 10–15 min of quiet rest in a seated position, each experimental testing session consisted of (1) instrumentation; (2) 10 min in room air for baseline; (3) 10 min with end-tidal partial CO_2_ pressure (PETCO_2_) clamped at 40 mmHg (cl-40); (4) 3 min of voluntary hyperventilation to lower PETCO_2_ to ~20 mmHg (HVE); and (5) a modified rebreathing test (REB, details below). Stages 3 to 5 of the protocol were carried out in a background of hyperoxia (end-tidal partial O_2_ pressure [PETO_2_] ~250 mmHg) so that the input from O_2_ chemoreceptors was reduced, so that the vast majority of the input would come from CO_2_ chemoreceptors. Clamping CO_2_ at 40 mmHg normalized the conditions to look at the influence of the peripheral chemoreflex. Rebreathing was used to provoke an incremental increase in CO_2_, consequently increasing BP, and thus bringing the baroreflex into play. Figure 1 shows a block-diagram of the resting protocol.

### Resting protocol experimental setup

Throughout the protocol, the subjects sat upright and breathed through a mouthpiece attached to a two-way, non-rebreathing valve (Hans-Rudolph 2700, Hans-Rudolph, Shawnee, KS, USA). The breathing circuit allowed switching from room air to either an end-tidal clamping system or a rebreathing system. The end-tidal clamping setup used in the present study was a modified version of the system previously described by Olin et al. (2012). The setup allowed stabilizing PETCO_2_ at 40 mmHg by constantly adding a varying portion of CO_2_ into the inspired gas mixture. Throughout the end-tidal PCO_2_ clamping, we maintained PETO_2_ at ~250 mmHg by titrating 50% (balanced with N_2_) or 100% O_2_ into the inspiratory reservoir, at SL and ALT, respectively.

### Modified rebreathing method

The rebreathing bag was filled with gas to achieve inspired PCO_2_ and PO_2_ of 0 mmHg and 300 mmHg, respectively, at each altitude. Subjects were instructed to hyperventilate for 3 min (*part 4*) to lower and then maintain PETCO_2_ at 20 mmHg at both sea level and 5,260 m (in a background PETO_2_ of ~250 mmHg). Subjects were then switched to the rebreathing bag and following two initial deep breaths to mix the gas from the bag with that in the respiratory system, they were instructed to breathe *ad libitum* (*part 5*). The rebreathing tests were terminated when PETCO_2_ reached 50 mmHg, PETO_2_ dropped below 200 mmHg, or the subject reached the end of his or her hypercapnia tolerance.

### Exercise protocol

**Figure 2:**
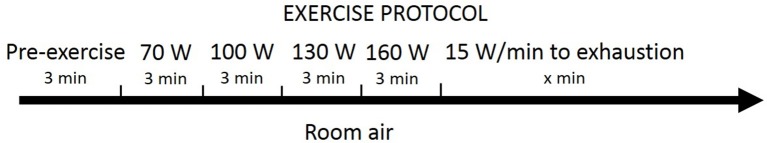
Block-diagram of the timeline for the exercise protocol.

Subjects were seated on an electrically-braked cycle ergometer (Velotron Elite, Racermate, Seattle, WA, USA). The protocol began with a three-min resting baseline in pedaling position on the ergometer. The subjects then completed four 3-min stages at 70, 100, 130, and 160 Watts, followed by 15 Watts/min increments until they could no longer maintain pedaling >50 rpm despite strong verbal encouragement. No specific pedaling frequency was required. Maximal power output (Watts) was calculated as: work rate of last stage completed + [(work rate increment) × (time into final stage/duration of stage, in seconds)] (Subudhi et al., 2008). We used the exercise paradigm to increase BP in a functional capacity. Subjects breathed room air throughout the exercise protocol. Figure 2 shows a block-diagram of the exercise protocol.

### Measurements

#### Arterial blood gas

During the exercise protocol, arterial blood samples (2 ml) were taken from the catheter in a radial artery during the resting baseline, at the end of each of the four three-min stages and immediately before the cessation of exercise (BL, 70W, 100W, 130W, 160W, and MAX). Core body temperature was telemetrically recorded from an ingested pill (CorTemp; HQInc, Palmetto, FL, USA) and used to correct the results from the blood gas analyzer. All samples were analyzed immediately for arterial PO_2_ (PaO_2_), PCO_2_ (PaCO_2_), and pHa in triplicate (Rapidlab 248; Siemens Healthcare Diagnostics, Munich, Germany). The blood gas analyzer was calibrated daily with tonometered whole blood samples.

#### Metabolic variables

Throughout the exercise protocol, the subjects breathed through a mouthpiece connected to a two-way, non-rebreathing valve (Hans-Rudolph 2700, Hans-Rudolph, Shawnee, KS, USA). Ventilation and respiratory frequency (Rf) were measured using a pneumotachograph (Universal Ventilation Meter; Vacu·Med, Ventura, CA, USA; Ultimaseries; Medgraphics CPX, Minneapolis, MN, USA), expressed in units adjusted to body temperature and pressure, saturated (BTPS) and averaged over windows of 30 s. PETO_2_ and PETCO_2_ were measured using fast-responding gas analyzers (O_2_Cap Oxygen analyzer; Oxigraf, Mountain View, CA, USA). The pneumotachograph was calibrated using a 3-liter syringe (Hans-Rudolph 5530, Shawnee, KS, USA) and the gas analyzers were calibrated using gas mixtures of known concentrations of O_2_ and CO_2_ prior to each testing session.

### Data acquisition

All analog data were sampled and recorded at 200 Hz on a personal computer for off-line analysis (Powerlab 16/30; ADInstruments, Bella Vista, Australia).

### Data analysis

Heart beat-to-beat time intervals were extracted directly from BP recordings. Initially, systolic blood pressure (SBP) peaks were extracted from the BP waveform with heartbeats representing the time of their occurrence. However, low sampling rates (<250 Hz) may produce jitter in the estimation of peaks (Merri et al., 1990; Task Force, 1996). For instance, at 200 Hz the highest time resolution is within a confidence interval of 5 ms. To refine the location of heartbeats and the SBP values, a second order polynomial was interpolated for each extracted peak using four neighbor samples from the BP waveform (two immediately before and two immediately after). Heartbeats were selected as the location of the maximum of the interpolated polynomial. Furthermore, SBP values were updated as the maximum in their corresponding polynomial. Finally, the inter-beat intervals (IBI) were created as the interval between successive peaks.

The BRS was then calculated using the most commonly used “sequence method,” providing a direct interpretation of the causal link between blood pressure and heart rate (Parati et al., 1988). This is the “gold standard” and most reliable method, with proven clinical value (Pinna et al., 2015). This method is based on the identification of at least three consecutive beats in which a strictly defined increase (or decrease) in SBP is followed by a strictly defined increase (or decrease) in the IBI. Fixed minimal changes were considered for SBP and IBI to validate a sequence. Specifically, a minimum change of 1 mmHg between two consecutive SBP values or of 5 ms for IBI was set as the smallest increase (or decrease) in a sequence (Bernardi et al., 2010). Furthermore, the minimum correlation coefficient between changes in SBP and IBI to validate a sequence was set at 0.85. Finally, a minimum number of five sequences was set to validate a BRS estimate. The sensitivity of the reflex is obtained by computing the slope of the regression line between changes in SBP and IBI. All computed slopes are finally averaged to obtain the BRS. The advantage of this method is that the computations are automatic and standardized, which virtually eliminates intra- and inter-subject measurement variability (La Rovere et al., 2008). The baroreflex nature of these spontaneous RR interval-systolic pressure sequences was demonstrated by showing that in cats the number of sequences markedly dropped (−89%) after the surgical opening of the baroreflex loop by sinoaortic denervation (Di Rienzo et al., 2001).

BRS was thus assessed using a 90-s window immediately before the termination of each resting intervention and exercise stage.

BRS depends on SBP and IBI fluctuations. However, respiration affects both SBP and IBI via mechanisms that are not necessarily of baroreflex origin. Whether, respiratory sinus arrhythmia is due to a central mechanism or to the baroreflex mechanism is debated (Eckberg, 2009; Karemaker, 2009). Previous work attempted to separate the effects of the baroreflex and respiration using metronome-guided respiration and adaptive filtering of the data (Tiinanen et al., 2008), and showed that the respiratory rate, but not the pattern is of primary importance (Paprika et al., 2014). Therefore, to control for a potential effect of hyperventilation on BRS in hypoxic conditions, respiration frequency was extracted via an autoregressive power spectral density (PSD) estimation of the IBI. The PSD was estimated with an order of 50, and the respiration frequency was extracted as that of the largest peak in the range [0.1–0.4] Hz. This IBI frequency band was larger than that of the conventional heart rate variability (HRV) high frequency band, i.e., [0.15–0.4] Hz, as respiration frequency can migrate to the low frequency band [0.04–0.15] Hz. BRS values with and without the respiratory frequency are reported in this work (Tables 4, 5).

### Statistics

**Figure 3:**
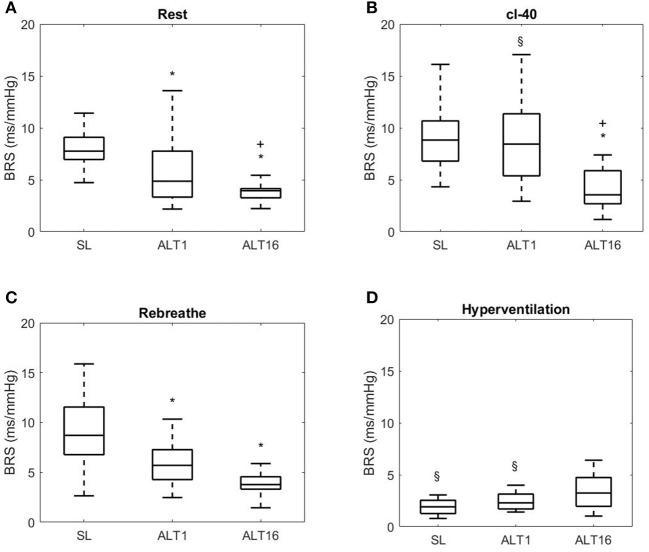
Tukey boxplots, horizontal line inside boxes: median; upper and lower lines of boxes: 75th and 25th percentiles, respectively; upper and lower whiskers: highest and lowest data points within the 1.5 inter quartile range. Outliers not shown. BRS, baroreflex sensitivity; SL, sea level; ALT1: 1st day at 5,260 m; ALT16: 16th day at 5,260 m. **(A)** Rest, breathing room air; **(B)** cl-40, end tidal CO_2_ clamped at 40 mmHg (cl-40). **(C)** Rebreathe, breathing in a close circuit, Reb (rising end tidal CO_2_ to 50 mmHg); **(D)** Hyperventilation, lowering end tidal CO_2_ to 20 mmHg (HVE). *Different from SL, + different from ALT1, § different from Rest.

Figures 3, 6 display Tukey boxplots of the data in which the horizontal line inside the boxes is the median, whilst the upper and lower lines of the boxes are the 75th and 25th percentiles, respectively. The upper and lower whiskers denote the highest and lowest data points within the 1.5 inter quartile range (Frigge et al., 1989). This corresponds to approximately ± 2.7σ and 99.3% coverage of the data (McGill et al., 1978). The outliers are not shown on Figures 3, 6 for scaling purposes.

Two-way repeated measures ANOVAs were performed to assess the effect of time (SL vs. ALT1 vs. ALT16) and the effect of condition (Rest vs. cl-40 vs. hyperventilation vs. rebreathe) in the rest protocol, and the effect of time and exercise intensity (PRE, 70W, 100W, 130W, 160W, and MAX) in the exercise protocol. One-way repeated measures ANOVA was performed to assess the effect of condition when time was not available (time decay). The Tukey-Kramer post hoc test was performed when appropriate. The alpha level for significance was set at 0.05 and is reported rounded to three digits after the decimal. All analyses were completed using MATLAB® (MathWorks, Natick, MA, USA). The coefficient of variation (CV) and the inferior and superior 95% confidence interval (CI_inf_ and CI_sup_, respectively) of BRS are reported in Table 2 for the rest protocol and Table 3 for the exercise protocol.

**Table 2:** Mean values ± SD during the rest protocol.

	**Rest**			**Clamp-40**			**REB**			**HVE**		
	***SL***	***ALT1***	***ALT16***	***SL***	***ALT1***	***ALT16***	***SL***	***ALT1***	***ALT16***	***SL***	***ALT1***	***ALT16***
meanBP	79 ± 7	75 ± 12	79 ± 11	82 ± 7	88^*^ ± 10	105^*^^+^ ± 11	91^a^ ± 10	291^a^^*^ ± 61	216^a^^*^^+^ ± 79	82 ± 10	83 ± 10	83 ± 9
PaO_2_	102.1 ± 5.2	35.7^*^ ± 3.1	44.9^*^^+^ ± 3.5	282.1^a^ ± 14.1	280.3^a^ ± 14.6	255.1^a^ ± 32.8	241.4^a^ ± 14.6	265.8^a^ ± 22.3	257.7^a^ ± 30.0	255.3^a^ ± 20.4	292.6^a^ ± 16.1	302.6^a^ ± 17.0
PaCO_2_	38.2 ± 4.2	26.7^*^ ± 3.1	21.1^*^^+^ ± 2.8	40.5^a^ ± 2.2	39.5^a^ ± 2.5	39.5^a^ ± 2.0	47.3^a^^b^ ± 1.7	50.8^a^^b^^*^ ± 2.0	50.9^a^^b^^*^ ± 4.2	17.4^a^^b^^c^ ± 2.4	16.9^a^^b^^c^ ± 2.5	18.1^a^^b^^c^ ± 2.5
pHa	7.408 ± 0.028	7.503^*^ ± 0.027	7.494^*^ ± 0.019	7.392 ± 0.021	7.389^a^ ± 0.035	7.330^a^^*^^+^ ± 0.022	7.360^a^^b^ ± 0.048	7.325^a^^b^^*^ ± 0.024	7.276^a^^b^^*^^+^ ± 0.030	7.631^a^^b^^c^ ± 0.042	7.618^a^^b^^c^ ± 0.040	7.529^a^^b^^c^^*^^+^ ± 0.036
Rf	0.270 ± 0.064	0.292 ± 0.057	0.272 ± 0.077	0.284 ± 0.057	0.329^a^ ± 0.053	0.493^a^^*^^+^ ± 0.141	0.301 ± 0.073	0.406^a^^b^^c^^*^ ± 0.073	0.580^a^^*^^+^ ± 0.147	0.329^a^ ± 0.047	0.335^a^ ± 0.006	0.288^b^^c^^*^^+^ ± 0.094
HR	76 ± 12	91^*^ ± 18	98^*^^+^ ± 13	74 ± 11	76^a^ ± 14	100^*^^+^ ± 18	71 ± 12	87^b^^*^ ± 17	100^*^^+^ ± 17	107^a^^b^^c^ ± 20	115^a^^b^^c^ ± 30	104 ± 15

*meanBP, mean arterial blood pressure (mmHg); PaO_2_, arterial oxygen partial pressure (mmHg); PaCO_2_, arterial carbon dioxide partial pressure (mmHg); pHa, arterial blood pH; Rf, respiratory frequency (Hz); HR, heartrate (bpm)*.

a *Significant compared to rest (p < 0.05)*.

b *Significant compared to clamp-40 (p < 0.05)*.

c Significant compared to REB (p < 0.05),

* significant compared to SL (p < 0.05),

+ *significant compared to ALT1 (p < 0.05)*.

**Table 3:** Mean values ± SD during the exercise protocol.

	**Rest**			**70W**			**100W**			**130W**			**160W**			**Max**		
	***SL***	***ALT1***	***ALT16***	***SL***	***ALT1***	***ALT16***	***SL***	***ALT1***	***ALT16***	***SL***	***ALT1***	***ALT16***	***SL***	***ALT1***	***ALT16***	***SL***	***ALT1***	***ALT16***
meanBP	95± 11	94± 9	92^a^± 9	101± 10	101± 10	101^a^± 14	106^a^± 10	103^a^± 9	104^a^± 9	109^a^^b^± 9	106^a^± 7	108^a^± 8	113^a^^b^± 8	106^a^^*^± 6	112^a^^b^^+^± 9	121^a^^b^^c^^d^± 8	109^a^^b^^*^± 10	110^a^^b^^*^± 11
PaO_2_	102± 7	40^*^± 4	48^a*^^+^± 5	95^a^± 5	33^a^^*^± 3	42^a^^*^^+^± 3	97± 5	32^a^^*^± 2	41^a^^*^^+^± 3	96^a^± 6	33^a^^*^± 3	41^a^^*^^+^± 3	96^a^± 7	33^a^^*^± 2	41^a^^*^^+^± 3	97^a^± 8	34^a^^*^± 2	42^a^^*^^+^± 4
PaCO_2_	36± 4	27^*^± 3	20^*^^+^± 3	38± 3	27^*^± 2	20^*^^+^± 2	39± 3	25^*^± 2	20^*^^+^± 2	38± 4	23^a^^b^^*^± 3	19^*^^+^± 2	37± 4	22^a^^b^^c^^*^± 2	17^a^^b^^c^^d^^*^^+^± 3	32^a^^b^^c^^d^^e^± 5	21^a^^b^^c^^d^^*^± 3	16^a^^b^^c^^d^^*^^+^± 2
pHa	7.421± 0.027	7.505^*^± 0.025	7.502^*^± 0.030	7.393± 0.016	7.486^*^± 0.021	7.472^a^^*^^+^± 0.022	7.380^a^± 0.020	7.460^a^^*^± 0.028	7.449^a^^*^± 0.032	7.367^a^± 0.031	7.429^a^^b^^c^^*^± 0.039	7.433^a^^b^^*^± 0.028	7.343^a^^b^^c^± 0.049	7.414^a^^b^^c^^*^± 0.034	7.411^a^^b^^c^^*^± 0.033	7.257^a^^b^^c^^d^^e^± 0.058	7.379^a^^b^^c^^d^^e^^*^± 0.044	7.369^a^^b^^c^^d^^e^^*^± 0.039
Rf	0.259± 0.065	0.297± 0.045	0.344^*^± 0.104	0.358± 0.087	0.419^a^± 0.076	0.457^*^± 0.123	0.391^a^± 0.098	.500^a^^*^± 0.106	0.545^a^^*^± 0.152	0.465^a^± 0.158	0.651^a^^b^^c^^*^± 0.128	0.665^a^^b^^*^± 0.123	0.579^a^^b^^c^± 0.241	0.791^a^^b^^c^^d^^*^± 0.132	0.791^a^^b^^c^^*^± 0.155	0.965^a^^b^^c^^d^^e^± 0.213	0.818^a^^b^^c^^d^± 0.234	0.946^a^^b^^c^^d^^e^± 0.261
HR	77± 17	90^*^± 12	97^*^± 15	108^a^± 18	135^a^^*^± 10	123^a^^*^^+^± 20	125^a^^b^± 21	149^a^^b^^*^± 11	138^a^^b^^*^^+^± 18	140^a^^b^^c^± 21	162^a^^b^^c^^*^± 9	149^a^^b^^c^^*^^+^± 13	155^a^^b^^c^^d^± 20	167^a^^b^^c^^d^^*^± 6	157^a^^b^^c^^d^^+^± 12	185^a^^b^^c^^d^^e^± 14	170^a^^b^^c^^d^^*^± 12	166^a^^b^^c^^d^^e^^*^± 13

*meanBP, mean arterial blood pressure (mmHg); PaO_2_, arterial oxygen partial pressure (mmHg); PaCO_2_, arterial carbon dioxide partial pressure (mmHg); pHa, arterial blood pH; Rf, respiratory frequency (Hz); HR, heartrate (bpm)*.

a *Significant compared to rest (p < 0.05)*.

b *Significant compared to 70 W (p < 0.05)*.

c *Significant compared to 100 W (p < 0.05)*.

d *Significant compared to 130 W (p < 0.05)*.

e *Significant compared to 160 W (p < 0.05)*,

* *significant compared to SL (p < 0.05)*,

+ *significant compared to ALT1 (p < 0.05)*.

## Results

Table 1 summarizes the subjects' characteristics.

### Resting protocol results

BRS decreased at ALT1 during seated rest (*p* = 0.048 vs. SL), this decrease was exacerbated at ALT16 (*p* < 0.001 vs. ALT1) as shown on Figure 3A.

During cl-40, BRS did not change compared to rest at SL (*p* = 0.499). BRS did not change between SL and ALT1 (*p* = 0.341) but decreased at ALT16 (*p* = 0.021 vs. SL and ALT1) as shown on Figure 3B. BRS at ALT1 increased compared to rest (*p* = 0.031 vs. rest ALT1) back to SL values. At ALT16, it did not change compared to rest (*p* = 0.213). In short, clamping CO_2_ at 40 mmHg did not affect BRS at SL whilst at ALT1 it restored it to SL values.

During REB, BRS decreased from SL to ALT1 (*p* = 0.002) and ALT16 (*p* = 0.002) but there was no difference between ALT1 and ALT16 (*p* = 0.792), as shown on Figure 3C. BRS did not change compared to rest in SL, ALT1, and ALT16.

No significant differences across time were found during HVE (all *p* > 0.050). At SL and at ALT1, there was a significant decrease in BRS during HVE compared to rest (both *p* < 0.001 vs. rest SL and ALT1) but not at ALT16 (*p* = 0.770).

Table 2 summarizes mean BP, PaO_2_, PaCO_2_, pHa, Rf, and HR values during rest, cl-40, REB and HVE at SL, ALT1, and ALT16.

**Figure 4:**
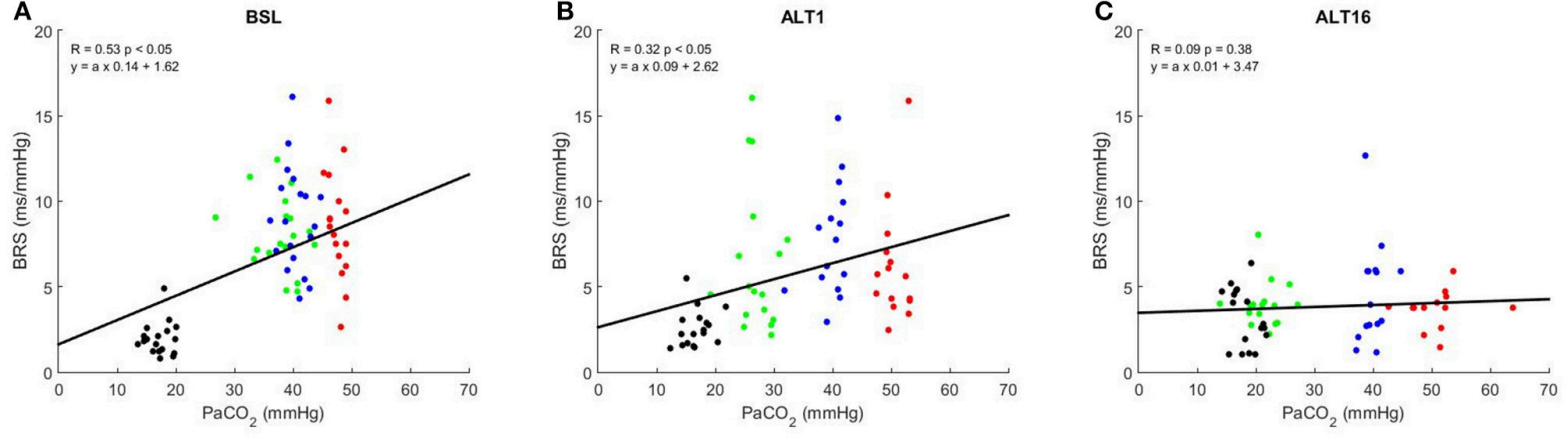
Correlation graphs of arterial partial pressure of CO_2_ (PaCO_2_) vs. baroreflex sensitivity (BRS). R Pearson's correlation coefficient. Green dots, breathing room air (Rest); blue dots, clamp 40 (cl-40); red dots, rebreathing (REB); black dots, hyperventilation (HVE). BSL: sea level, **(A)**; ALT1: 1st day at 5,260 m, **(B)**; ALT16: 16th day at 5,260 m, **(C)**.

**Figure 5:**
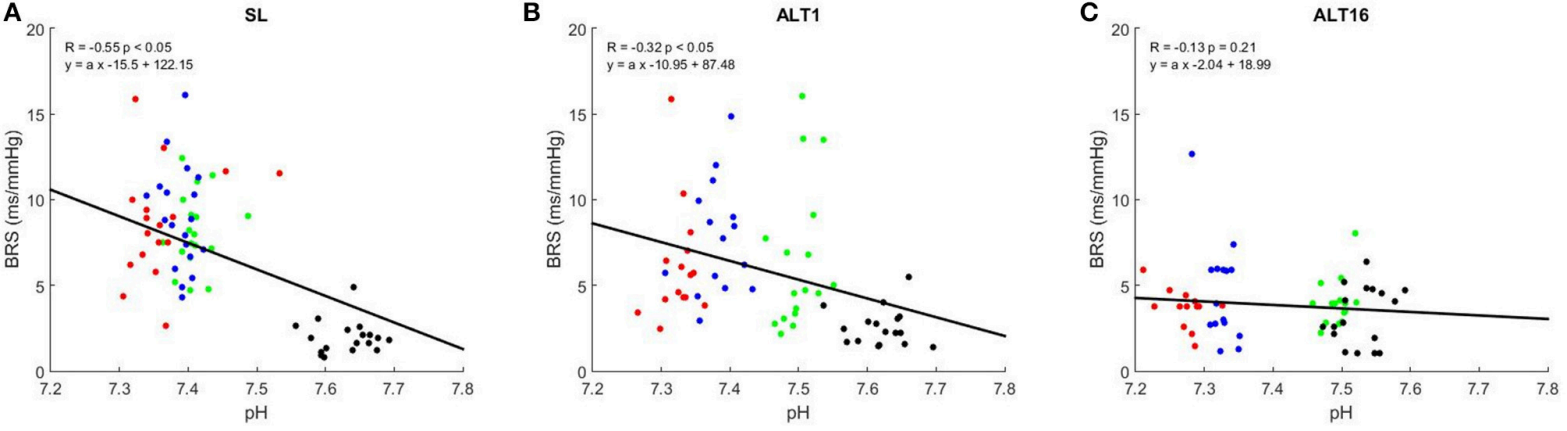
Correlation graphs of arterial blood pHa vs. baroreflex sensitivity (BRS). R Pearson's correlation coefficient. Green dots, breathing room air (Rest); blue dots, clamp 40 (cl-40); red dots, rebreathing (REB); black dots, hyperventilation (HVE). SL: sea level, **(A)**; ALT1: 1st day at 5,260 m, **(B)**; ALT16: 16th day at 5,260 m, **(C)**.

Figure 4 shows the correlation graphs between PaCO_2_ and BRS at SL, ALT1, and ALT16. The slope of this relationship decreased from SL (0.22 ± 0.10) to ALT1 (0.12 ± 0.10, *p* < 0.001 vs. SL) and further to ALT16 (0.03 ± 0.06, *p* < 0.001 vs. ALT1). An inverse relationship was found between pHa and BRS. As illustrated on Figure 5, the slope flattened from SL (−23.2 ± 11.0) to ALT1 (−14.6 ± 11.1, *p* < 0.001 vs. SL) and further to ALT16 (−4.1 ± 7.9, *p* < 0.001 vs. ALT1).

### Exercise protocol results

**Figure 6:**
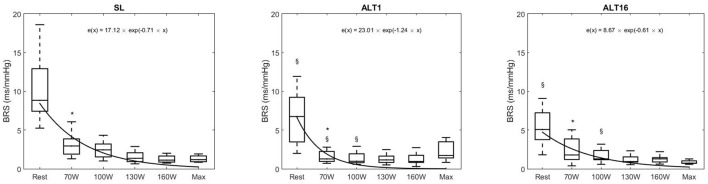
Tukey boxplots of baroreflex sensitivity (BRS) during the exercise protocol to exhaustion, horizontal line inside boxes: median; upper and lower lines of boxes: 75th and 25th percentiles, respectively; upper and lower whiskers: highest and lowest data points within the 1.5 inter quartile range. Outliers not shown. BRS at rest, 70, 100, 130, 160 Watts and maximal exercise. SL, sea level; ALT1, 1st day at 5,260 m; ALT16, 16th day at 5,260 m. black curves are mono-exponential fit of the medians of the exercise intensities. *Different from rest (*p* < 0.05), § different from SL (*p* < 0.05).

Figure 6 shows the reduction in BRS when exercise intensity increased at SL, ALT1, and ALT16, which was expected. This reduction was fitted with a mono-exponential and revealed that the time decay was lower at ALT1 compared to SL (*p* = 0.003) and ALT16 (*p* = 0.004). Time decay was not different between SL and ALT16 (*p* = 0.718). These results indicate that the kinetics of the decrease in BRS when exercise intensity increases were not different between SL and ALT16 although the pre-exercise BRS value was lower in ALT16 compared to SL (*p* = 0.002). At ALT1, the kinetics were different compared to SL and ALT16, notably because of a greater decrease between pre-exercise and 70W and a BRS roughly stable at the subsequent workloads. The decrease in BRS during exercise appeared to be more progressive during SL and ALT16 than during ALT1.

At SL, ALT1, and ALT16, BRS decreased between pre-exercise and 70W (*p* = 0.001, *p* = 0.032, *p* = 0.021, respectively). The decrease was not significant between the subsequent exercise stages (all *p* > 0.050). Pre-exercise BRS decreased from SL to ALT1 (*p* = 0.003) and to ALT16 (*p* = 0.003); there was a tendency between ALT1 and ALT16 (*p* = 0.083). BRS at 70W decreased at ALT1 compared to SL (*p* = 0.001) and ALT16 (*p* = 0.016), but there was no significant difference between SL and ALT16 (*p* = 0.125). BRS at 100W decreased from SL to ALT1 (*p* = 0.035) and ALT16 (*p* = 0.025), whilst there was no significant difference between ALT1 and ALT16 (*p* = 0.682). At 130W, 160W, and Max, there were no differences between SL, ALT1, and ALT16 (all *p* > 0.050).

Table 3 summarizes mean BP, PaO_2_, PaCO_2_, pHa, Rf, and HR values during rest, 70W, 100W, 130W, 160W, and Max at SL, ALT1, and ALT16.

### Respiration effects on BRS

Table 4 summarizes BRS assessed with and without the respiratory frequency during the rest protocol. Only the differences between BRS with respiration and BRS without respiration are reported. For BRS differences between the experimental conditions, please refer to Figure 3. Removing respiration decreased BRS at SL, ALT1, and ALT16 during rest (*p* < 0.001, *p* = 0.048, *p* = 0.003, respectively); at SL and ALT16 during cl-40 (*p* < 0.001, *p* < 0.001, respectively); at SL, ALT1 and ALT16 during REB (*p* < 0.001, *p* = 0.002, *p* < 0.001, respectively); and at SL during HVE (*p* = 0.002). BRS also decreased at ALT1 and ALT16 compared to SL after removal of respiration.

**Table 4:** BRS during the rest protocol with and without respiratory frequency. CV and CI refer to BRS with respiration.

	**Rest**			**cl-40**			**REB**			**HVE**		
	***SL***	***ALT1***	***ALT16***	***SL***	***ALT1***	***ALT16***	***SL***	***ALT1***	***ALT16***	***SL***	***ALT1***	***ALT16***
BRS – resp (ms/mmHg)	8.1 ± 1.9	6.5 ± 3.6	4.0 ± 1.1	9.0 ± 2.7	10.4 ± 7.9	5.4 ± 4.8	10.1 ± 6.0	7.0 ± 4.0	6.6 ± 9.4	2.7 ± 3.1	3.5 ± 4.0	3.2 ± 1.4
BRS – no resp (ms/mmHg)	3.9 ± 1.9^*^	2.9 ± 1.0^*^^a^	2.3 ± 0.4^*^^a^	4.6 ± 3.6^*^	5.0 ± 1.9^§^	4.1 ± 4.7^*^	3.9 ± 1.9^*^	5.0 ± 1.8^*^^§^	4.3 ± 2.0^*^^§^	4.5 ± 2.4^*^	2.5 ± 1.0^a^	2.4 ± 1.5^a^
CV	0.23	0.55	0.27	0.30	0.27	0.89	0.59	0.57	1.41	1.13	1.12	0.44
CI_inf_	7.3	5.0	3.5	7.8	7.1	3.4	7.6	5.3	2.7	1.4	1.9	2.6
CI_sup_	8.9	8.0	4.5	10.1	13.8	7.4	12.6	8.7	10.6	4.1	5.2	3.9

BRS – resp, baroreflex sensitivity assessed using the sequence method (ms/mmHg); BRS – no resp, baroreflex sensitivity assessed using the sequence method with respiration frequency removed (ms/mmHg);

* *significant compared to BRS - resp (p < 0.05)*.

§ *different from rest (p < 0.05)*.

a * Different from SL (p < 0.05). Coefficient of variation (CV), superior (CI_sup_), and inferior (CI_inf_) confidence interval boundaries of BRS (ms/mmHg) with respiration. The CI values have been computed using the classical 95% confidence interval*.

Table 5 summarizes BRS assessed with and without the respiratory frequency during the exercise protocol. Only the differences between BRS with respiration and BRS without respiration are reported. For BRS decrease with exercise and hypoxia, please refer to Figure 6. At rest and 70W, removing respiration decreased BRS at SL and ALT1 (*p* < 0.001, *p* = 0.003, *p* = 0.022, *p* = 0.001, respectively). At 100W, 130W, 160W and Max, removing respiration did not significantly alter BRS (all *p* > 0.050).

**Table 5:** BRS during exercise with and without respiratory frequency.

	**Rest**			**70W**			**100W**			**130W**			**160W**			**Max**		
	***SL***	***ALT1***	***ALT16***	***SL***	***ALT1***	***ALT16***	***SL***	***ALT1***	***ALT16***	***SL***	***ALT1***	***ALT16***	***SL***	***ALT1***	***ALT16***	***SL***	***ALT1***	***ALT16***
BRS – resp (ms/mmHg)	11.1± 6.3	7.1± 4.1	5.3± 2.1	3.3± 1.7	1.5± 0.7	2.4± 1.4	2.6± 1.4	1.6± 1.0	1.7± 0.7	1.7± 1.2	2.0± 2.1	1.7± 1.8	2.1± 2.8	1.3± 0.5	1.3± 0.3	4.5± 9.6	5.9± 6.7	0.8± 0.2
BRS – no resp (ms/mmHg)	6.1± 3.1^*^	3.2± 2.5^*^^a^	3.1± 0.3^a^	2.2± 1.0^*^^§^	1.4± 0.3^*^^§^^a^	1.7± 0.4^§^	2.1± 0.7^§^	1.7± 0.3^§^	1.8± 0.5^§^	1.6± 0.6^§^	2.0± 1.2^§^	1.4± 0.2^§^	3.5± 3.1	2.1± 0.7	1.3± 0.3^§^	3.8± 2.8^§^	4.6± 3.1	1.2± 1.4^§^
CV	0.57	0.58	0.41	0.50	0.43	0.59	0.53	0.67	0.44	0.69	1.09	1.04	1.33	0.42	0.26	2.15	1.14	0.19
CI_inf_	7.8	4.9	4.1	2.4	1.2	1.7	1.9	1.0	1.3	1.1	0.8	0.8	0.6	1.0	1.1	−0.7	2.3	0.8
CI_sup_	14.5	9.3	6.4	4.2	1.9	3.2	3.4	2.1	2.1	2.4	3.1	2.7	3.6	1.5	1.5	9.6	9.4	0.9

CV and CI refer to BRS with respiration. BRS – resp, baroreflex sensitivity assessed using the sequence method (ms/mmHg); BRS – no resp, baroreflex sensitivity assessed using the sequence method with respiration frequency removed (ms/mmHg);

* *significant compared to BRS - resp (p < 0.05)*.

§ *different from rest (p < 0.05)*.

a *Different from SL (p < 0.05). Coefficient of variation (CV), superior (CI_sup_), and inferior (CI_inf_) confidence interval boundaries of BRS (ms/mmHg) with respiration. The CI values have been computed using the classical 95% confidence interval*.

## Discussion

This study is the first to report BRS systematically, during acute and chronic exposure to 5,260 m, at rest and during dynamic exercise to exhaustion. The main findings were (1) BRS decreased in acute hypoxia and this decrease was exacerbated after acclimatization to hypoxia; (2) the decrease in acute hypoxia was reversible when clamping end-tidal CO_2_ at 40 mmHg, but not after acclimatization to hypoxia; and (3) the decrease in BRS during exercise was greater at low exercise intensities only, in acute hypoxia compared to normoxia; after acclimatization to hypoxia, the decrease in BRS during exercise returned toward SL values.

### BRS at rest

The decrease in BRS at rest in acute hypoxia confirmed previous findings in the literature (Roche et al., 2002) but the amplification of this decrease after acclimatization is a new finding. The background of hyperoxia reduced the input from the O_2_ chemoreceptors, therefore leaving the majority of the input of the chemoreflex to CO_2_ receptors. The immediate reversal of the decrease in BRS during cl-40 at ALT1 (return to SL values) indicated that reduced CO_2_ is likely the main trigger for BRS decrease in acute hypoxia. The reduction in BRS in acute hypoxia is probably partially mediated by the carotid body chemoreceptors (Mozer et al., 2016) even though hyperoxia greatly diminishes their contribution. Previous studies suggested that acute hypoxia initiates a persistent increase in chemoafferent activity to the rostroventrolateral medulla via the nucleus tractus solitarius, which results in long-lasting sympathoexcitation (Guyenet, 2000; Prabhakar and Kumar, 2010). Hypocapnia deactivated the chemoreceptors which resulted in decreased BRS (Querido et al., 2011; Tremblay et al., 2016), whilst normalizing CO_2_ during cl-40 at ALT1 restored BRS to SL values because the sensitivity of the chemoreceptors was unchanged (no resetting).

In chronic hypoxia, additional inspired CO_2_ had very little effect if any on BRS (cl-40 at ALT16, Figure 3) which might be associated with an enhanced carotid body hypoxic sensitivity (Tatsumi et al., 1991). Accordingly, enhanced chemosensory and ventilatory responses in chronic hypoxia are thought to induce a significant decrease in BRS (Del Rio et al., 2014), which the present results seem to confirm although it was carried out in a hyperoxic background. Contribution of the peripheral chemoreceptors may be enhanced with acclimatization but remain minor compared to contribution of the central chemoreceptors. Also, the present findings are consistent with a previous report from the AltitudeOmics expedition (i.e., same participants during the same experiment) demonstrating that there is a resetting of the cerebrovascular CO_2_ reactivity operating point to lower PaCO_2_ following acclimatization to high altitude (Fan et al., 2015). This cerebrovascular resetting is probably the result of an altered acid-base buffer status resulting from prolonged exposure to the severe hypocapnia associated with ventilatory acclimatization. Finally, a reduction in beta-adrenergic cardiac sensitivity with acclimatization (Richalet et al., 1990) might also have contributed to decrease BRS by potentially reducing the response of the heart to the afferent commands from the chemoreflex arc.

The present experiments were conducted in a background of hyperoxia, such that the chemoreceptors sensitive to O_2_ were silenced. Therefore, the changes in BRS described during cl-40, REB and HVE are mostly due to the CO_2_ sensitive central and peripheral chemoreceptors. Also, the central chemoreceptors are known to be more responsive to CO_2_ than the peripheral ones (Dempsey et al., 2014; Smith et al., 2015), hence the aforementioned mechanisms are principally due to the central chemoreceptors and for a minor part to the peripheral chemoreceptors.

In normoxia HVE toward a PETCO_2_ of 20 mmHg decreased BRS. This was probably because of the resultant decreased PaCO_2_. This effect was also found in acute hypoxia, albeit with a lower amplitude (Figure 3). In chronic hypoxia, PaCO_2_ during HVE did not change compared to rest and therefore did not affect BRS. PaCO_2_ levels during HVE were similar in SL, ALT1, and ALT16, and so were the BRS, but probably through different mechanisms as suggested in the previous paragraphs. Taken together these results suggest that the CO_2_ chemoreceptors play a pivotal role in BRS reduction in SL and ALT1 during HVE, whilst BRS resetting at ALT16 may explain the little effect of HVE. Prolonged exposure to PaCO_2_ as low as 20 mmHg likely provoked a resetting of the CO_2_ chemoreceptors around this value. Suddenly returning PaCO_2_ to 40 mmHg triggers severe hyperventilation and is hardly tolerated, however it does not restore BRS (condition cl-40), therefore evidencing the reset of the CO_2_ chemoreceptors toward low PaCO_2_.

Rebreathing did not affect BRS but provoked large increases in BP at ALT1 and ALT16, as if the capacity of the baroreflex was overwhelmed by the very high levels of CO_2_ and concomitant rapid drop in blood pHa. To be able to correct such changes in BP, BRS should have augmented by a very large margin, probably more than it is physically able to do, hence the enormous increase in BP. Additionally, in hypoxic conditions BRS decreases, which is not in favor of the ability to correct large increases in BP.

BRS decreases when HR increases. Intrinsic HR does not seem to change significantly with acute hypoxia (i.e., no change in the face of combined adrenergic and vagal blockade) suggesting that the ANS mechanisms must be responsible for HR (and BRS) changes at altitude (Koller et al., 1988).

Our results can be summarized as follows: (1) the rest condition shows BRS with low O_2_ and CO_2_ levels; (2) The Clamp-40 condition normalizes CO_2_, however the respiratory frequency is increased, which we address further in a dedicated section; (3) The REB condition shows BRS with high levels of CO_2_ and increased respiratory frequency; (4) Finally, the HVE condition shows BRS with low levels of CO_2_ and an effect from central command on breathing pattern. The results obtained in these conditions clearly show the effects of CO_2_ on the chemoreflex, but cannot exclude a partial role of respiratory frequency, tidal volume (lung stretch) or central command. A previous study tightly controlling ventilation for tidal volume and respiratory frequency showed that hyperpnoea did not influence BRS whilst hypoxia and hypocapnia did (Halliwill et al., 2003). The present report shows that chronic exposure to altitude provokes an adaptation of BRS (responses to CO_2_ are changed) which is independent of the hypoxic and hypocapnic conditions inherent to altitude exposure (e.g., normalizing CO_2_ in cl-40 in chronic altitude did not restore BRS to SL values).

Similar PaCO_2_ were targeted for hypo and hypercapnic conditions in SL, ALT1, and ALT16, for example making cl-40 a normocapnic condition in SL but a hypercapnic condition in ALT1 and ALT16. The goal of this experimental design was to demonstrate an absolute reset of the chemoreceptors after acclimatization. That is, after prolonged exposure to low PaCO_2_, the CO_2_ chemoreceptors activity would be centered on a low PaCO_2_. Suddenly returning to a PaCO_2_ of 40 mmHg immediately restores BRS in ALT1 therefore indicating no resetting, whilst the same sudden exposure at ALT16 does not restore BRS to SL value indicating a resetting. Choosing what values to target is not easy. Depending on the question asked, different targets would be appropriate, for example, using same delta PaCO2 from normocapnia in each condition (Rupp et al., 2013), could have led to changes in sensitivity i.e., different changes in BRS to a given delta of PaCO_2_ but would have prevented the demonstration of the absolute reset. Therefore, comparing different conditions (i.e., normocapnia vs. hpercapnia), was the only way to demonstrate the absolute reset.

Plotting BRS against the wide range of PaCO_2_ available in the present study allows us to assess how much the baroreflex can control BP from HVE to REB, notably by taking the slope of this relationship, which can be used to assess baroreflex gain. Figure 4 shows that this gain significantly decreased from SL to ALT1 and further to ALT16, a finding consistent with the decrease in sensitivity and which is probably dependent on the same mechanisms. In acute and particularly in chronic hypoxia, the baroreflex partially loses its ability to control BP when PaCO_2_ changes. Accordingly, a previous study showed that respiratory-induced variations in blood pressure are greater in hypoxic conditions (Brown et al., 2014), suggesting an impaired control of blood pressure. The inverse relationship was found when BRS was plotted vs. pHa, but the physiological principle remains the same. The central chemoreceptors are excited in acute hypoxia and their sensitivity is enhanced in chronic hypoxia, which results in a decrease in BRS gain and ability to properly control BP over a wide range of PaCO_2_ (Fan et al., 2014).

### BRS during exercise

Baroreflex resetting during exercise occurred at SL, ALT1, and ALT16 as we found no major changes in BP during exercise. However, BRS during exercise seemed more affected in ALT1 than in ALT16. Indeed, at SL and ALT16 the decrease in BRS with increasing exercise intensity was progressive (similar time decays) whilst in ALT1 there was a sudden drop between rest and 70W, BRS remaining around its 70W value for the subsequent exercise stages (changed time decay, Figure 6). This observation suggests that the acute hypoxia-induced heightened activation of the chemoreceptors affects the resetting of the baroreflex. The enhanced sensitivity of the chemoreceptors occurring with acclimatization then seemed to let BRS resetting recover toward SL levels, the progressive decrease in BRS during exercise being comparable between SL and ALT16, the main difference being at rest.

### Influence of the respiratory frequency on BRS

One of the main physiological factors affecting BRS is the respiratory frequency (Horsman et al., 2015). In the present study, hypoxia *per se* did not modify the respiratory frequency (Table 2) and accordingly, removing the respiratory frequency from the blood pressure signal decreased BRS in SL, ALT1, and ALT16 (Table 4). Therefore, the decrease in BRS at rest in ALT1 and ALT16 is not due to variations in the respiratory frequency. During cl-40, the respiratory frequency increased at ALT1 and ALT16 because of the increased ventilatory drive from high CO_2_. However, when removing the respiratory frequency, the comparisons between SL, ALT1 and ALT16 did not change much and this would therefore not affect our interpretation of the data. During REB, removing the respiratory frequency abolished the differences between SL, ALT1, and ALT16 and increases BRS compared to rest in ALT1 and ALT16 (Table 4). However, the corresponding mean BPs are very high (291 and 216 mmHg respectively, Table 3). Therefore, interpretation of BRS should be made with caution. BP regulation is designed to work around mean BP of about 100, or 130–140 at maximum, certainly not above 200 mmHg. During HVE removing the respiratory frequency diminished BRS in SL but not in ALT1 and ALT16. PaCO_2_ during HVE did not change in ALT1 and ALT16 (i.e., HVE is normocapnic) whilst it greatly diminished at SL (i.e., HVE is hypocapnic) therefore decreasing BRS. Even if removing respiration modified BRS response in HVE, we still contend that PaCO_2_ is essential, whilst respiratory frequency probably comes as a second factor. Overall it seems that the effects of the rest protocol on BRS were mainly due to modifications occurring in the arc reflex controlling the baroreflex rather than to an artifact linked to the respiratory frequency.

During the exercise protocol, removing the respiratory frequency decreased BRS at rest and 70W only. This decrease would not affect our interpretation of the results since the differences between SL, ALT1, and ALT16 remained consistent. From 100W to Max, removing the respiration frequency did not significantly change BRS.

## Limitations

In our setup there was no independent control of PO_2_ and PCO_2_, i.e., interventions which modified inspired O_2_ only or modified inspired CO_2_ only. Therefore, we cannot entirely identify the influence of each and we cannot exclude that the diminishing hyperoxic background triggered a light hyperventilation toward the end of our experimental sequence, due to O_2_ chemoreceptors (Querido et al., 2010). However, our study setting allowed to determine that CO_2_ has a greater relative importance as compared to O_2_ in the BRS reduction in acute and chronic hypoxia, which is concordant with previously published studies.

The technique used to define and detect the limits between pulse cycles influences the resulting time series of pulse-to-pulse intervals (Schäfer and Vagedes, 2013). IBI detection using the timing of the systolic peak as fiducial point to measure IBI may be imprecise (Suhrbier et al., 2006). Numerous studies are based on algorithms for detecting systolic peaks, which make our study comparable to the literature. Additionally, pulse rate variability or heart rate variability analyses require high precision in IBI detection over several minutes of recordings. In the BRS analysis, we are only looking for a difference between two consecutive IBI of more or less than 5 ms to validate a sequence. Therefore, the time precision is of lesser importance compared to other types of analysis. Finally, we used intra-arterial catheterization to obtain the traces of continuous blood pressure. Therefore, the signal is clean (low noise) and better delineated than in the great majority of studies, where photo-plethysmography originated signals were recorded. Therefore the technique presently used (interpolation coupled to maximum finding) is of good quality.

The evaluation of BRS is an established tool for the assessment of autonomic control of the cardiovascular system. Besides the well-acknowledged physiological role in the maintenance of circulatory homeostasis, evidence has been accumulating that changes in the characteristics of baroreflex function reflect alterations in autonomic control of the cardiovascular system (Eckberg and Sleight, 1992). Measuring the baroreflex has been shown to be a source of valuable information in the clinical management of a variety of diseases (La Rovere et al., 2008). However, studying the baroreflex responses in humans exposed to high altitude is difficult. The neck chamber system has been proposed (Parati and Mancia, 1992; Sagawa et al., 1997) to study BRS under lowered carotid transmural pressure (therefore simulating hypobaria), but the spontaneous BRS method during a prolonged sojourn probably remains the closest to reflecting *in vivo* natural adaptation of the cardiovascular system to high altitude (Roche et al., 2002). The aim of this study was to investigate the spontaneous variations in BRS, which includes its manifestation occurring with respiration. Removing respiration was an enormous work but provides a view as comprehensive as possible to the readers. It is evident that removing respiration flattens the continuous BP signal, hence removing many occurrences of BP sequences to be included in the computations, therefore constituting an artificial BP (i.e., variations in BP are never free of influence from respiration). Similarly, HR increased at altitude, which may decrease BRS. It is unfortunately not possible to estimate what would have been BRS if HR had not changed. Yet, our results demonstrate that CO_2_ is essential in BRS adaptations during acclimatization to high altitude.

Other methods exist to estimate BRS (such as the Transfer Function method and Bernardi's ratio of the standard deviations). Comparable trends between those methods have been reported (Laude et al., 2004). Applied on our dataset, Bernardi's method gave results close to the sequence method and interpretation of our results would not have been different (data not shown). The Transfer Function method showed more discrepant results and aberrant values. Detailed differences between those methods on our dataset were beyond the scope of the present article.

Also, using the sequence method, positive and negative sequences were isolated and treated separately, which did not significantly change the results. When more than three consecutive points constituting a sequence were found BRS was calculated with and without overlap of those points, which again did not significantly change the results. The sequence method has a number of limits concerning the criteria about what sequences can be used, but we have taken as many precautions as we could to ensure that the reported BRS represent as fairly as possible what is actually going on during acclimatization to altitude.

Finally, neural and molecular mechanisms of the BRS reduction in acute and chronic hypoxia were also beyond the scope of the present work. Future studies are needed to further explore the mechanisms.

## Conclusions

This study is the first to report a decrease in BRS in acute hypoxia that was exacerbated after acclimatization to 5,260 m. Because this decrease in acute hypoxia was reversible when clamping CO_2_ at sea level values in acute hypoxia, but not in chronic hypoxia, we contend the following mechanisms to explain our findings: during acute exposure, a hypoxia-induced heightened activation of the CO_2_ chemoreceptors would be the main trigger, whilst in chronic hypoxia an increase in CO_2_ chemoreceptor sensitivity might be the principal cause of the attenuated BRS. The increased sensitivity during acclimatization is not immediately reversible with acute hyperoxia. Future studies, using a tight control of the respiratory pattern, are needed to determine the nature of the neural and molecular changes underlying those decreases in BRS. During incremental exercise, the BRS decrease is similar in normoxia and chronic hypoxia whilst it happens at lower intensity and with larger drop in acute hypoxia.

## Author contributions

AL, AS, BK, and RR conceived and designed the experiments. NB and AS performed the experiments NB, SY, and J-MV analyzed data. NB interpreted the data, wrote the first version of the manuscript, and prepared the figures. SY, AS, AL, RR, J-MV, and BK revised the manuscript. All authors approved final version of the manuscript.

### Conflict of interest statement

The authors declare that the research was conducted in the absence of any commercial or financial relationships that could be construed as a potential conflict of interest.
